# Improved Chambadal Model with New Optimization Results

**DOI:** 10.3390/e26020125

**Published:** 2024-01-31

**Authors:** Michel Feidt, Monica Costea

**Affiliations:** 1 Laboratory of Energetics, Theoretical and Applied Mechanics (LEMTA), URA CNRS 7563, University of Lorraine, 54518 Vandoeuvre-lès-Nancy, France; michel.feidt@univ-lorraine.fr; 2 Department of Engineering Thermodynamics, National University of Science and Technology POLITEHNICA Bucharest, 060042 Bucharest, Romania

**Keywords:** Carnot irreversible engine, Chambadal model, optimization, mechanical energy, power, entropy production, energy degradation

## Abstract

This paper presents a continuation of the Chambadal model optimization of the irreversible Carnot engine. We retrieved the results presented in the Special Issue “Carnot Cycle and Heat Engine Fundamentals and Applications II” and enriched them with new contributions that allowed comparing two points of view: (1) the now classical one, centered on entropy production in the four processes of the cycle, which introduces the action of entropy production, with several sequential optimizations; (2) the new one that is relative to an energy degradation approach. The same démarche of sequential optimization was used, but the results were slightly different. We estimate that the second approach is more representative of physics by emphasizing the energy conservation and the existence on an upper and a lower bound in the mechanical energy and power output of the engine.

## 1. Introduction

The year 2024 marks the two-century anniversary of Carnot booklet publication [1]. Although the well-known maximum efficiency of the cycle introduced by Carnot is relative to equilibrium conditions, and the source and the sink are thermostats (infinite heat reservoirs) at, respectively, *T_HS_* (hot source) and *T_CS_* (cols sink), this cycle is still used.

Since that publication, numerous papers related to the Carnot cycle have been published. Among them, we noticed the paper of Curzon and Ahlborn [2], which proposes an expression of efficiency associated with the first law that was derived at maximum work *W* and maximum power output $\dot{W}$ of the Carnot engine, in endo-reversible conditions (or no conversion irreversibilities):

(1) $$\eta_{I\;endo}\left(MaxW\right)=1-\sqrt{\frac{T_{CS}}{T_{HS}}}$$

This first limitation of the Carnot cycle efficiency was particularly reconsidered in a previous Special Issue [3] and in [4] from a historical point of view.

The topic of efficiency at maximum power was widely developed by scientists in the last 50 years, since recent research in Web of Science (January 2024) using this keyword found 32,221 papers. Among them, 2076 publications are relative to *engine* efficiency at maximum power, and four of them were published in 2023.

We noted an important evolution in these works, starting with classical configurations of engines [5] and evolving towards quantum engines and statistical modeling [6]. However, recent studies on classical engines [7,8] and quantum engines [9] concern other configurations than the Carnot one, since they are oriented towards applications rather than theoretical aspects.

Another recent research trend is the scale reduction of engines. As a proof, many studies are devoted to a quantum approach [9,10], small-scale models [11], or biomolecular configurations [12]. They show that improvements are always possible.

Back to the Carnot cycle and its improvements, we noted that the main recent contributions focus on the study of the effect of the cycle’s irreversibilities on its performance.

Thus, Ref. [4] confirmed a natural gradation of the models, from Carnot [13], through Chambadal [3,14], to Curzon-Ahlborn, by taking into account transfer and conversion irreversibilities.

To summarize, two ways have been mainly used to introduce irreversibilities in a model: (1) the ratio method, proposed by Ibrahim et al. [15] and Novikov [16]; (2) the entropy production method [17]. Our choice fell on the last method, which was used throughout this study.

In 2019, we published the first paper [3] relative to progress in the Carnot and Chambadal modeling of thermomechanical engines by considering entropy production and heat transfer entropy.

It was pursued in 2022 by another paper [18] that was integrated in the second Special Issue “Carnot Cycle and Heat Engines Fundamentals and Applications II”.

We propose here for the third Special Issue “Carnot Cycle and Heat Engines Fundamentals and Applications III” a revisited Chambadal model of the Carnot engine, providing new results.

It appears that the year 2024, marking the two-century anniversary of the Carnot booklet publication, will offer surprises because of the many additions and extensions of the Carnot cycle modeling that are still possible and are on course.

Section 2 provides a summary of the results obtained previously for the Chambadal configuration [18]. It presents a first optimization relative to *T_H_* (the hot-side temperature of the cycle) with a linear heat transfer law, and a second one relative to transformation duration. Afterward, an optimization of the mean power with respect to the cycle duration is described.

Section 3 reconsiders the irreversible Chambadal engine based on a degraded energy point of view instead of an entropy production one, as presented in Section 2.

Section 4 presents a comparison–discussion of the results described in Section 2 and Section 3.

Section 5 reports conclusions and perspectives.

## 2. Chambadal Model Optimization Based on Entropy Production and with a Coupling Constraint for the Heat Transfer between Source and Converter

**Figure 1:**
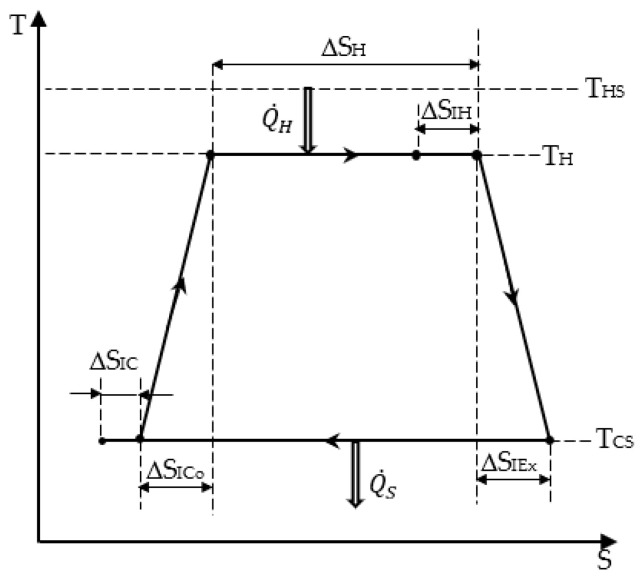
Chambadal irreversible model.

We summarize hereafter the results obtained in the preceding paper [18]. According to Figure 1, we have:

- The heat energy expense from the hot source that is expressed as:

(2) $$Q_{H}=T_{H}∆S_{H},$$

where *T_H_* is the hot temperature of the cycle, and Δ*S_H_* is the heat transfer entropy at the source.

- The heat energy converted into mechanical one along the isothermal transformation at *T_H_*:

(3) $$Q_{conv}=T_{H}\left(∆S_{H}-∆S_{IH}\right),$$

where Δ*S_IH_* is the entropy production in the hot temperature isothermal transformation of the cycle.

- The entropy balance over the cycle, expressed by:

(4) $$∆S_{conv}+∆S_{I}=∆S_{S},$$

with Δ*S_S_* being the entropy rejected ($Q_{S}=T_{CS}∆S_{S}$) during the isothermal transformation at *T_CS_*, generally with *T_CS_* = *T*_0_, i.e., ambient temperature.

- The total entropy production of the cycle Δ*S_I_*, which is the sum of the four entropy productions during the processes:

(5) $$∆S_{I}=∆S_{IH}+∆S_{IEx}+∆S_{IC}+∆S_{ICo}.$$

- The energy balance over the cycle for the system composed of heat source, converter, and heat sink:

(6) $${W=Q}_{conv}-Q_{S}.$$

By combining Equations (3) and (5) with Equation (6), we obtain:

(7) $$W=\left(T_{H}-T_{CS}\right)∆S-T_{H}∆S_{IH}-T_{CS}\left(∆S_{IEx}+∆S_{IC}+∆S_{ICo}\right),$$

where the notation Δ*S_H_* = Δ*S* defines the reference heat transfer entropy. It corresponds to the heat expense.

We note that the reference entropy Δ*S* implies an endo-reversible mechanical energy that only retains the first term of Equation (7), that is:

(8) $$W_{endo}=\left(T_{H}-T_{CS}\right)∆S,$$

And the entropy production along the adiabatic processes is associated with *T_CS_*. In fact, this corresponds to a maximum of *W*, because Δ*S_IEx_* and Δ*S_ICo_* are produced within the [*T_CS_*, *T_H_*] range.

Also, it appears that the heat energy expense from the hot source (Equation (2)) can be expressed as depending on the heat transfer conductance $G_{H}$ [3]:

(9) $$Q_{H}=T_{H}∆S=\left(T_{HS}-T_{H}\right)G_{H}.$$

Consequently, as Δ*S* and *G_H_* are considered parameters of the cycle (converter) and the source, respectively, the temperature *T_H_* results to be:

(10) $$T_{H}=\frac{G_{H}T_{HS}}{G_{H}+∆S}.$$

This result differs from those of the modified Chambadal engine [18], according to which we obtained:

(11) $$T_{H}^{*}=\sqrt{\frac{T_{HS}T_{CS}}{1+s_{I}}},$$

with $s_{I}=\frac{∆S_{IH}}{G_{H}}$ being a specific ratio relative to the irreversible isothermal transformation *T_H_*, and

(12) $$Max_{1}W=G_{H}{\left(\sqrt{T_{HS}}-\sqrt{\left(1+s_{I}\right)T_{CS}}\right)}^{2}-T_{CS}∆S_{I}.$$

The subsequent optimization is relative to the transformation duration, with $∆S_{Ii}=\frac{C_{Ii}}{\tau_{i}}$. According to published results that were reported in Appendix B in [18] and are relative to *Max_2_W*:

(13) $$Max_{2}W\approx W_{endo}-\frac{T_{CS\;}}{\tau }N^{2},$$

with ${\tau_{H}}^{*}=\sqrt{\sqrt{T_{0}T_{HS}}\frac{C_{IH}}{\lambda }}$, and ${\tau_{i}}^{*}=\sqrt{\frac{{T_{0}C}_{Ii}}{\lambda }}$; $\sqrt{\lambda }=\frac{N\sqrt{T_{HS}}}{\tau }$.

*W_endo_* is deduced from Equation (11) and corresponds to Δ*S_I_* = 0.

This second energy optimization is completed by the mean power optimization over the cycle period *τ*, knowing that $N=\sqrt{\frac{T_{CS}}{T_{HS}}C_{IH}}+\sqrt{C_{IEx}}+\sqrt{C_{IC}}+\sqrt{C_{ICo}}.$ We obtain:

(14) $$\tau^{*}=\frac{{2T}_{CS}N^{2}}{W_{endo}}.$$

The main power over a cycle is:

(15) $$Max\bar{\dot{W}}\approx \frac{{W_{endo}}^{2}}{4T_{CS}N^{2}},$$

and the corresponding efficiency could be determined, though within the scope of the approximation indicated by Equation (13). We propose here to reconsider the optimization without this approximation.

## 3. Chambadal Model Optimization from the Energy Degradation Point of View

We introduced energy degradation in each transformation of the cycle and globally as:

(16) $$E_{I}=E_{IH}+E_{IEx}+E_{IC}+E_{ICo},$$

where *E_I_* is the total energy degradation considered as a parameter, and *E_Ii_* is the energy degradation in process *i* of the cycle.

By using Equation (7) and the relation of energy degradation in each process, $\frac{E_{Ii}}{T_{i}}=∆S_{Ii}$, we obtain:

(17) $$W=\left[G_{H}\left(T_{HS}-T_{H}\right)-E_{IH}\right]\left(1-\frac{T_{CS}}{T_{H}}\right)-T_{CS}\left(\frac{E_{IH}}{T_{H}}+\frac{E_{IEx}}{T_{IEx}}+\frac{E_{IC}}{T_{CS}}+\frac{E_{ICo}}{T_{ICo}}\right).$$

We noticed that the mechanical energy output from the cycle depends on the four degradations of mechanical energy *E_Ii_* and on the four corresponding temperatures. The two temperatures of the isothermal transformations are known (*T_H_* and *T_CS_*), but the temperatures of the adiabatic transformations depend on the irreversibility path of the cycle.

Figure 1 illustrates all available options. Among them, we noticed that for the two adiabatic processes, the corresponding temperatures pertain to the range [*T_CS_*, *T_H_*].

Thus, if we choose in Equation (17) the first limiting case *T_IEx_* = *T_ICo_* = *T_H_*, we obtain the lowest value of the ratios $\frac{E_{IEx}}{T_{IEx}}$ and $\frac{E_{ICo}}{T_{ICo}}$ in the two adiabatic transformations and, correspondingly, the highest value of the mechanical energy output, *Sup W*.

For the second limiting case *T_IEx_* = *T_ICo_* = *T_HS_* in Equation (17), the effect will be quite opposite, determining the highest value of the two ratios $\frac{E_{IEx}}{T_{IEx}}$ and $\frac{E_{ICo}}{T_{ICo}}$, and the lowest useful effect, *INF W*.

Consequently, an upper bound and a lower bound for W yields, from Equation (17):

(18) $$SUP\;W=\left[G_{H}\left(T_{HS}-T_{H}\right)-E_{IH}\right]\left(1-\frac{T_{CS}}{T_{H}}\right)-T_{CS}\left(\frac{E_{IH}}{T_{H}}+\frac{E_{IEx}}{T_{H}}+\frac{E_{IC}}{T_{CS}}+\frac{E_{ICo}}{T_{H}}\right),$$

(19) $$INF\;W=\left[G_{H}\left(T_{HS}-T_{H}\right)-E_{IH}\right]\left(1-\frac{T_{CS}}{T_{H}}\right)-T_{CS}\left(\frac{E_{IH}}{T_{H}}+\frac{E_{IEx}+E_{IC}+E_{ICo}}{T_{CS}}\right).$$

After some arrangements, the resulting expression of Equation (18) is:

(20) $$SUP\;W=G_{H}\left(T_{HS}-T_{H}\right)\left(1-\frac{T_{CS}}{T_{H}}\right)-T_{CS}\left(\frac{E_{IH}}{T_{CS}}+\frac{E_{IC}}{T_{CS}}+\frac{E_{IEx}+E_{ICo}}{T_{H}}\right),$$

and Equation (19) combined with Equation (16) leads to:

(21) $$INF\;W=G_{H}\left(T_{HS}-T_{H}\right)\left(1-\frac{T_{CS}}{T_{H}}\right)-E_{I}.$$

Based on Equations (20) and (21), *Max*_1_*(SUP W)* and *Max*_1_*(INF W)* can be determined (see Appendix A). We obtained the following expressions of the maximum mechanical energy delivered by the cycle:

(22) $$Max_{1}\left(SUP\;W\right)=G_{H}{\left(\sqrt{T_{HS}+\frac{E_{IA}}{G_{H}}}-\sqrt{T_{CS}}\right)}^{2}-E_{I},$$

(23) $$Max_{1}\left(INF\;W\right)=G_{H}{\left(\sqrt{T_{HS}}-\sqrt{T_{CS}}\right)}^{2}-E_{I}.$$

The corresponding efficiency at maximum mechanical energy yields:

(24) $$\eta \left(Max_{1}SUP\;W\right)=\frac{{\left(\sqrt{G_{H}T_{HS}+E_{IA}}-\sqrt{{G_{H}T}_{CS}}\right)}^{2}-E_{I}}{\sqrt{G_{H}T_{HS}+E_{IA}}\left(\sqrt{G_{H}T_{HS}+E_{IA}}-\sqrt{{G_{H}T}_{CS}}\right)-E_{IA}},$$

(25) $$\eta \left(Max_{1}INF\;W\right)=\frac{G_{H}{\left(\sqrt{T_{HS}}-\sqrt{T_{CS}}\right)}^{2}-E_{I}}{G_{H}\sqrt{T_{HS}}\left(\sqrt{T_{HS}}-\sqrt{T_{CS}}\right)}=1-\sqrt{\frac{T_{CS}}{T_{HS}}}-\frac{E_{I}}{G_{H}\sqrt{T_{HS}}\left(\sqrt{T_{HS}}-\sqrt{T_{CS}}\right)}.$$

It is easy to see in the above results (Equation (22) or Equation (23)) that we recovered the maximum of the mechanical work in the endo-reversible case as a limit, when the energy degradation in all transformations was zero (*E_Ii_* = 0, see Equation (16)):

(26) $$MaxW_{endo}=G_{H}{\left(\sqrt{T_{HS}}-\sqrt{T_{CS}}\right)}^{2}.$$

The cycle efficiency corresponding to *Max W_endo_* retrieves from Equation (24) or Equation (25) the well-known expression:

(27) $$\eta \left(MaxW_{endo}\right)=1-\sqrt{\frac{T_{CS}}{T_{HS}}}.$$

## 4. Discussion

### 4.1. Model Considering Heat Transfer Entropy

In a previous paper [18], the reference heat transfer entropy corresponded to heat expense (Δ*S_H_* = Δ*S*), but without the coupling constraint between the heat source and the converter. Consequently, the optimization of mechanical energy was performed with respect to the temperature *T_H_* (variable), as originally proposed by Chambadal, though in the endo-reversible case (Section 2):

(28) $$T_{H}^{*}=\sqrt{\frac{T_{HS}T_{CS}}{1+s_{I}}},$$

with $s_{I}=\frac{∆S_{IH}}{G_{H}}$, i.e., specific entropy ratio.

The maximum of the mechanical energy yields:

(29) $$max_{1}W=G_{H}{\left(\sqrt{T_{HS}}–\sqrt{\left({1+s}_{I}\right)T_{CS}}\right)}^{2}-T_{0}∆S_{I}.$$

A sequential optimization was performed using a simple form of entropy production in each process of the cycle, expressed by:

(30) $$∆S_{Ii}=\frac{C_{Ii}}{\tau_{i}}.$$

and introducing the new concept of *entropy production action*, *C_I_*, expressed in Js/K [13].

According to Equation (30), *C_Ii_* is a quantity related to the entropy production in each process of the cycle. This new concept was introduced by analogy to the well-known action principle of Maupertuis [19], reason why we chose to call it *action of entropy production* related to the irreversibility of process *i*. Until now, it was considered a constant parameter without physical significance.

Thus, the duration of each process associated with the entropy production action (Equation (30)) submitted to a finite cycle duration constraint led to a second sequential optimization. The entropy method allowed obtaining *Max*_2_*W* in an approximated form corresponding to the low-irreversibility case in a *T_H_* isothermal process. The results of this optimization were:

- The optimum duration of the isothermal process at *T_H_*:

(31) $${\tau_{H}}^{*}=\frac{\tau }{N}\sqrt{{\frac{T_{CS}}{T_{HS}}C}_{IH}}.$$

- The optimum duration of each process of the cycle:

(32) $${\tau_{i}}^{*}=\frac{\tau }{N}\sqrt{C_{Ii}}.$$

- The maximum of the mechanical energy:

(33) $$Max_{2}W\approx W_{endo}-\frac{T_{CS\;}}{\tau }N^{2},$$

with $N=\sqrt{\frac{T_{CS}}{T_{HS}}C_{IH}}+\sqrt{C_{IEx}}+\sqrt{C_{IC}}+\sqrt{C_{ICo}}.$

A third optimization regarding, this time, the power output of the engine was performed, generating the following result:

(34) $$\bar{\dot{W}}\left(Max_{2}W\right)=\frac{{W_{endo}}^{2}}{4T_{CS}N^{2}},$$

for $\tau^{*}=\frac{{2T}_{CS}N^{2}}{W_{endo}}.$

### 4.2. Model Considering Energy Degradation

In the present paper (Section 3), we developed a model that considers the cycle irreversibilities from the energy degradation point of view, instead of the entropy production perspective, in each process. However, these two irreversibilities are related by $∆S_{Ii}=\frac{E_{Ii}}{T_{i}}$.

We noticed in this relation that, for the two isothermal transformations, the temperatures *T_H_* and *T_CS_* were known, while for the two adiabatic processes, the temperatures *T_IEx_* and *T_ICo_* depended on their irreversibility path, though they were always in the [*T_CS_, T_H_*] range.

The result was that, for the first time to our knowledge, an upper and a lower bound for the mechanical energy output in the irreversible case could be derived:

(35) $$SUP\;W=G_{H}\left(T_{HS}-T_{H}\right)\left(1-\frac{T_{CS}}{T_{H}}\right)-T_{CS}\left(\frac{E_{IH}}{T_{CS}}+\frac{E_{IC}}{T_{CS}}+\frac{E_{IEx}+E_{ICo}}{T_{H}}\right),$$

(36) $$INF\;W=G_{H}\left(T_{HS}-T_{H}\right)\left(1-\frac{T_{CS}}{T_{H}}\right)-E_{I}.$$

The optimization of the mechanical energy in the two cases led to:

(37) $$Max_{1}SUP\;W={\left(\sqrt{G_{H}T_{HS}+E_{IA}}-\sqrt{G_{H}T_{CS}}\right)}^{2}-E_{I},$$

with ${T_{H}}^{*}=\sqrt{T_{HS}T_{CS}-\frac{{T_{CS}E}_{IA}}{G_{H}}}$, and $E_{IA}=E_{IEx}+E_{ICo}$.

(38) $$Max_{1}INF\;W=G_{H}{\left(\sqrt{T_{HS}}-\sqrt{T_{CS}}\right)}^{2}-E_{I},$$

with ${T_{H}}^{*}=\sqrt{T_{HS}T_{CS}}$.

The efficiencies corresponding to the maximum mechanical energy in the two cases are:

(39) $$\eta \left(MaxSUP\;W\right)=\frac{{\left(\sqrt{G_{H}T_{HS}+E_{IA}}-\sqrt{{G_{H}T}_{CS}}\right)}^{2}-E_{I}}{\sqrt{G_{H}T_{HS}+E_{IA}}\left(\sqrt{G_{H}T_{HS}+E_{IA}}-\sqrt{{G_{H}T}_{SC}}\right)-E_{IA}},$$

(40) $$\eta \left(MaxINF\;W\right)=\frac{G_{H}{\left(\sqrt{T_{HS}}-\sqrt{T_{CS}}\right)}^{2}-E_{I}}{G_{H}\left(T_{HS}-\sqrt{{T_{HS}T}_{CS}}\right)}.$$

## 5. Conclusions

The two models presented here are complementary.

The first one is based on an entropy approach and allows the optimization of mechanical energy and power output according to the entropy production model. Nevertheless, it remains to determine the equivalent temperature in each irreversible adiabatic process.

The second model, using the energy degradation approach, allows the optimization of the mechanical energy of the irreversible engine with known upper and lower bounds. It remains to extend it to the power optimization.

In the first case, entropy production is expressed in a simple form related to the duration of the processes:

$$∆S_{Ii}=\frac{C_{Ii}}{\tau_{i}}.$$

Thus, we introduced a new concept, namely, the *action of entropy production C_Ii_*, and the corresponding connection to time (second law of thermodynamics).

In the second case, energy degradation is expressed in relation to entropy production, as:

$$∆S_{Ii}=\frac{E_{Ii}}{T_{i}},$$

where *E_Ii_* is the mechanical energy degraded in process *i*. It relates directly to energy conservation (first law of thermodynamics).

For each case, the constraint of finiteness can be applied as follows:

1. First case, $\sum_{i}∆S_{Ii}=∆S_{I}$: entropy production method.
2. Second case, $\sum_{i}E_{Ii}=E_{I}$: energy degradation method.

The difference could explain the complementarity of the two approaches. Further developments are on course.

Two centuries have passed since the publication of Carnot’s booklet, which was the first step in the evolution of thermodynamics. Most probably, it was also the most difficult step because it introduced new concepts.

Now we see that we can pursue and complete this foresighted work by Carnot and enrich it by considering the irreversibilities during the four processes of the cycle.

## Data Availability

Data supporting reported results are available from the authors.

## Appendix A

**Optimization of the mechanical work *SUP W***

Equation (20) can be rewritten as:

(A1) $$SUP\;W=G_{H}\left(T_{HS}-T_{H}\right)\left(1-\frac{T_{CS}}{T_{H}}\right)-T_{CS}\left(\frac{E_{IA}}{T_{H}}+\frac{E_{IT}}{T_{CS}}\right),$$

where *E_IA_* = *E_IEx_* + *E_ICo_* is the energy degradation in the adiabatic processes, *E_IT_* = *E_IH_* + *E_IC_* is the energy degradation in the isothermal processes, and *E_I_* = *E_IA_* + *E_IT_* is the total energy degradation in the cycle.

The maximum *SUP W* is obtained by differentiation according to the variable *T_H_*:

(A2) $$\frac{\partial \left(SUP\;W\right)}{\partial T_{H}}=0=G_{H}\left[-1+\frac{T_{CS}}{T_{H}}+\left(T_{HS}-T_{H}\right)\frac{T_{CS}}{T_{H}^{2}}\right]+\frac{T_{CS}}{T_{H}^{2}}E_{IA}.$$

After some arrangements, a second-order equation in *T_H_* results in:

(A3) $$G_{H}T_{H}^{2}=G_{H}T_{HS}T_{CS}+E_{IA}T_{CS},$$

which leads to the only possible physical solution, expressed as:

(A4) $$T_{H}^{*}=\sqrt{\frac{G_{H}T_{HS}T_{CS}+E_{IA}T_{CS}}{G_{H}}}.$$

Once the optimum $T_{H}^{*}$ expression is found, it can be replaced in Equation (A1):

(A5) $$Max_{1}SUP\;W=G_{H}\left(T_{HS}-T_{H}^{*}\right)\left(1-\frac{T_{CS}}{T_{H}^{*}}\right)-E_{IT}-\frac{T_{CS}}{T_{H}^{*}}E_{IA}.$$

Furthermore, an arrangement of the terms is performed through the addition and subtraction of *E_IA_*:

(A6) $$Max_{1}SUP\;W=G_{H}\left(T_{HS}-T_{H}^{*}\right)\left(1-\frac{T_{CS}}{T_{H}^{*}}\right)-E_{IT}-E_{IA}+E_{IA}\left(1-\frac{T_{CS}}{T_{H}^{*}}\right),$$

which leads to a more compact expression of *MaxSUP W*:

(A7) $$Max_{1}SUP\;W=\left(G_{H}T_{HS}+E_{IA}-{G_{H}T}_{H}^{*}\right)\left(1-\frac{T_{CS}}{T_{H}^{*}}\right)-E_{I}.$$

By replacing Equation (A4) in Equation (A7) and after some calculations, we obtain:

(A8) $$Max_{1}SUP\;W=G_{H}{\left(\sqrt{T_{HS}+\frac{E_{IA}}{G_{H}}}-\sqrt{T_{CS}}\right)}^{2}-E_{I}.$$

**Optimization of the mechanical work *INF W***

The starting point in this optimization is:

(A9) $$INF\;W=G_{H}\left(T_{HS}-T_{H}\right)\left(1-\frac{T_{CS}}{T_{H}}\right)-E_{I},$$

which is differentiated according to the variable *T_H_*:

(A10) $$\frac{\partial \left(INF\;W\right)}{\partial T_{H}}=0=G_{H}\left[-1+\frac{T_{CS}}{T_{H}}+\left(T_{HS}-T_{H}\right)\frac{T_{CS}}{T_{H}^{2}}\right].$$

The only solution of Equation (A10) is:

(A11) $$T_{H}^{*}=\sqrt{T_{HS}T_{CS}}.$$

By replacing Equation (A11) in Equation (A9), we obtain:

(A12) $$Max_{1}INF\;W=G_{H}\left(T_{HS}-\sqrt{T_{HS}T_{CS}}\right)\left(1-\frac{T_{CS}}{\sqrt{T_{HS}T_{CS}}}\right)-E_{I}.$$

After some calculations, the final expression of *MaxINF W* is:

(A13) $$Max_{1}INF\;W=G_{H}{\left(\sqrt{T_{HS}}-\sqrt{T_{CS}}\right)}^{2}-E_{I}.$$

Note that the efficiencies corresponding to *Max_1_SUP W* and *Max_1_INF W* are provided by Equations (24) and (25) in the text.
